# The association between maternal diabetes and the risk of attention deficit/hyperactivity disorder in offspring: Updated systematic review and meta-analysis.

**DOI:** 10.1192/j.eurpsy.2024.317

**Published:** 2024-08-27

**Authors:** Y. D. Sinishaw, B. A. Dachew, G. Ayano, K. Betts, R. Alati

**Affiliations:** University, Curtin, Perth, Australia

## Abstract

**Introduction:**

The existing body of evidence on the association between maternal diabetes and attention deficit/hyperactivity disorder (ADHD) in offspring is inconsistent and inconclusive. Thus, we need to synthesise the available evidence to examine the association between maternal diabetes and risk of ADHD in offspring.

**Objectives:**

The aim of this meta-analysis was to examine the association between maternal diabetes and the risk of ADHD in offspring.

**Methods:**

We conducted a comprehensive search across PubMed, MEDLINE, EMBASE, Scopus, CINAHL and PsychINFO databases from their inception to September 8th, 2023. The methodological quality of the included studies was evaluated using Joanna Briggs Institute (JBI) and Newcastle-Ottawa Scale (NOS). Between-study heterogeneity was assessed using I2 statistic and potential publication bias was checked using both funnel plot and Egger’s test. Randomeffect model was used to calculate the pooled effect estimates and subgroup, sensitivity, and meta-regression were further performed to support our findings

**Results:**

Twenty observational studies (two cross-sectional, five case-control and thirteen cohort studies) were included in this systematic review and meta-analysis. Our meta-analysis indicated that intra-uterine exposure to any type of maternal diabetes was associated with an increased risk ADHD in offspring [RR=1.33: 95 % CI: 1.23–1.43, I2=79.9%]. When we stratified the analysis by maternal diabetes type, we found 17%, and 37% higher risk of ADHD in offspring exposed to maternal gestational [RR=1.17: 95 % CI: 1.07–1.29] and pre-existing diabetes [RR=1.37: 95 % CI: 1.27–1.48] compared to unexposed offspring respectively. Results of subgroup and sensitivity analysis further supported the robustness of our main finding.

**Conclusions:**

Our review suggested that exposure to maternal diabetes increased the risk of ADHD in offspring. These findings underscore the need for early screening and prompt interventions for exposed offspring.

**Disclosure of Interest:**

None Declared

